# Weaving the nest: extracellular matrix roles in pre-metastatic niche formation

**DOI:** 10.3389/fonc.2023.1163786

**Published:** 2023-06-07

**Authors:** Laura Patras, Doru Paul, Irina R. Matei

**Affiliations:** ^1^Children’s Cancer and Blood Foundation Laboratories, Department of Pediatrics, Division of Hematology/Oncology, Drukier Institute for Children’s Health, Meyer Cancer Center, Weill Cornell Medicine, New York, NY, United States; ^2^Department of Molecular Biology and Biotechnology, Center of Systems Biology, Biodiversity and Bioresources, Faculty of Biology and Geology, Babes-Bolyai University, Cluj-Napoca, Romania; ^3^Division of Hematology/Oncology, Department of Medicine, Weill Cornell Medicine, New York, NY, United States

**Keywords:** pre-metastatic niche, extracellular matrix, systemic disease, extracellular vesicles, cancer metastasis

## Abstract

The discovery that primary tumors condition distant organ sites of future metastasis for seeding by disseminating tumor cells through a process described as the pre-metastatic niche (PMN) formation revolutionized our understanding of cancer progression and opened new avenues for therapeutic interventions. Given the inherent inefficiency of metastasis, PMN generation is crucial to ensure the survival of rare tumor cells in the otherwise hostile environments of metastatic organs. Early on, it was recognized that preparing the “soil” of the distal organ to support the outgrowth of metastatic cells is the initiating event in PMN development, achieved through the remodeling of the organ’s extracellular matrix (ECM). Remote restructuring of ECM at future sites of metastasis under the influence of primary tumor-secreted factors is an iterative process orchestrated through the crosstalk between resident stromal cells, such as fibroblasts, epithelial and endothelial cells, and recruited innate immune cells. In this review, we will explore the ECM changes, cellular effectors, and the mechanisms of ECM remodeling throughout PMN progression, as well as its impact on shaping the PMN and ultimately promoting metastasis. Moreover, we highlight the clinical and translational implications of PMN ECM changes and opportunities for therapeutically targeting the ECM to hinder PMN formation.

## Introduction

The extracellular matrix (ECM) is a complex three-dimensional extracellular network surrounding and supporting all cells and tissues in the body. Although fundamentally composed of water, proteins, and polysaccharides, the ECM is highly heterogeneous, with a tissue-specific physical, topological, and biochemical composition generated during tissue development through dynamic bidirectional interactions between cells (epithelial, fibroblast, adipocyte, and endothelial) and their microenvironment (1). The ECM provides a structural foundation for cells within a tissue and ensures mechanical integrity. The molecular cues provided by the ECM modulate intercellular interactions, cell behavior, vascular development, and immune function, ultimately orchestrating tissue development, phenotype, and homeostasis (1–3).

## ECM functions in normal physiology

### The matrisome

The core matrisome in mammals comprises around 300 proteins with specific functions, mainly macromolecules such as proteoglycans (PGs) (compressibility), fibrous proteins such as collagens (tensile strength), elastins (elasticity), fibronectins (FNs) (stiffness), glycoproteins and laminins (cell adhesion) (1, 4–6), and ECM-associated proteins known as matricellular proteins (e.g., tenascin C, thrombospondin-1 (TSP-1), periostin (POSTN), osteopontin, and osteonectin, also known as secreted protein acidic and rich in cysteine (SPARC)) (7–10), without a primary structural role (reviewed in 3). ECM constituents, PGs, glycosaminoglycans (GAGs), and fibrous proteins assemble into solid gel scaffolds with distinct biochemical and biomechanical properties, which are dynamically remodeled and tailored depending on the specific physiological structure and functions of the tissue (e.g., bone loose connective tissue and blood plasma) (5, 11, 12). Tissue-specific differences in ECM organization and structure (13, 14) are ensured by variations in ECM composition, isoform expression (15, 16), alternative splicing (17), and post-translational modifications (PTMs) such as glycosylation (13, 14) that shape the functional outcome of ECM–cellular interactions.

### ECM architecture

ECM architecture consists of two distinct but interconnected structures: the basement membrane (BM), a condensed thin matrix lining the basal side of epithelial and endothelial tissues (18, 19), and the interstitial matrix (IM), an interlaced porous structure characteristic of interstitial connective tissues, which is the preponderant ECM (20, 21) (**Table 1**). The BM consists primarily of collagen IV and laminins, which confer resistance to tensile force, as well as entactin/nidogen and heparan sulfate (HS) proteoglycans (31, 70–72). Critical for vascular function, the BM regulates endothelial barrier function and cellular transmigration across the endothelium, while modular PGs such as perlecan, agrin, and collagen type XVIII (endostatin precursor) regulate angiogenesis (73–75). Moreover, the BM enforces tissue compartmentalization while conveying extracellular microenvironment messages to epithelial cells or endothelial cells and pericytes in blood vessels (14, 76). The IM is primarily assembled from collagens (e.g., type I and III), which, together with fibronectin, provide a structural foundation for tissue function and mechanical integrity, protecting the tissue by acting as a compression buffer against mechanical stress (77). Tenascin and anionic proteoglycans maintain tissue hydration and the pH of the local microenvironment (1, 5, 11, 17), while physiological crosslinking of the matrix maintains its integrity and provides resistance to proteolytic degradation (13, 54).

**Table 1:** ECM evolution during cancer progression.

ECM changes	Physiological ECM	Cancer-associated ECM	The pre-metastatic ECM	References
ECM determinants	Tissue-resident stroma or resident/infiltrating immune cells, quiescent and metabolically inactive fibroblasts are the primary source of ECM molecules (collagens, PGs, elastins, and GFs) and proteolytic enzymes (MMPs) driving physiological ECM degradation	Pro-tumorigenic, metabolically active CAFs, peritumoral fibroblasts, reactive stroma fibroblasts, and myofibroblasts are the major source of ECM molecules. CAFs secrete high levels of collagen I, FNs, laminin, and HA, increasing matrix stiffness. Cancer cells and immune cells also contribute to ECM changes.	PMN ECM remodeling by resident stroma and immune cells: - Lung via activation and differentiation into α-SMA myofibroblast or CAF phenotype - Bone fibroblast-like cells - Liver hepatocytes, hepatic stellate cells (HStCs) in the liver - LN fibroblastic reticular cells - Omentum preadipocytes and endothelial cells - Immune resident (macrophages in the liver, microglia in the brain) and infiltrating (MDSCs and inflammation-activated neutrophils) cells	(22–30)
Composition	Tissue-specific composition of diverse glycans (PGs and GAGs) and ECM proteins (collagen, elastin, fibronectin, and laminin) present in physiological ratios HA is an abundant component of the brain ECM, while collagen is less prevalent, and FN is concentrated around the BM	Increased ECM deposition of collagen (types I, III, and IV), FNs, HA, and altered levels of elastin, as well as PGs (decorin, biglycan, lumican, and fibromodulin) depending on the cancer type Increased TNC impacts tumor growth and metastasis by modulating the interactions of integrins and syndecans with FNs.	Increased FN deposition in lung, liver, and LN PMNs - Lung: fibronectin- or laminin-rich (S100A4+ fibroblasts); transcriptional regulation for CD44, osteopontin (OPN), collagens, and MMPs -liver: increased collagen I deposition - lung and bone: local LOX disrupts ECM homeostasis -omentum: increased collagen and adhesion molecules -brain: increased HA	(1, 5, 11, 31–39)
Organization, stiffness, and mechanical properties	Highly organized structures with tissue-specific fiber alignments and crosslinking Maintenance of the physiological balance between stiffness and elasticity by FNs and elastins Compliant and flexible ECM - Bone: mineralization of type I collagen resulting in increased strengths) with areas of dense or porous ECM - Brain: maintenance of BBB integrity	Disorganized ECM Irregular fiber alignments Excessive crosslinking, stiffness ECM is a scaffold for EC migration and tube formation Increased desmoplasia (growth of fibrous and connective tissue) through the upregulation of LOX enzymes	Altered architecture (increased orthogonal characteristics) Crosslinking of collagen type I and IV by LOX family enzymes, adhesion platform for BMDC infiltration Increased stiffness in the (lung, liver, and LN PMNs) and pro-fibrotic ECM determined by the increased secretion of FNs, collagen, and LOX enzymes that crosslink collagen Altered mechanics perturb the function of mechanosensitive cells Bone—osteogenic environment with bone lesions that promote bone colonization. Osteoclast-mediated bone resorption and osteolytic metastases Brain vascular remodeling and BBB barrier disruption promote angiogenesis	(4, 6, 24, 28, 29, 35, 40–47)
Cellular signaling	Abnormal Provides physiological signaling cues that regulate cell behavior (e.g., proliferation, differentiation, migration)	Abnormal ECM signaling Release of stored GFs, activation of GF-mediated cellular functions (TGF-β, VEGF, PDGF, EGF, bFGF, and HGF), promote inflammation, angiogenesis HA interactions promote anti-apoptotic pathways, EMT, and invasiveness	Lung-altered POSTN promotes WNT signaling in the lung PMN - FN-rich areas anchor integrin a4b1^+^/VEGFR1^+^ HPCs; VCAN deposition in the lungs and promotion of MET Altered integrin interactions with PMN ECM drive tumor colonization and thrombosis	(1, 48–51b; 52, 53)
Proteolysis	Balanced levels of proteases degrade and modify the ECM depending on tissue requirements	Overexpression of ECM remodeling enzymes Increased secretion and activation of enzymes that degrade the ECM: MMPs (MMP-1, MMP-2, MMP-9, and others), ADAMs (serine and threonine proteases), proteases released by neutrophils, NET formation Dysregulated proteolysis leading to excessive ECM degradation and remodeling, enhanced tumor angiogenesis, invasion, and metastasis	Proteolytic remodeling by MMPs, MT MMPs, cathepsins, serine protease inhibitors - Lung: EV-mediated MMP-2 and MMP-9 production, which cleaves collagen IV; TIMP-1-mediated cleavage of MMPs - Neutrophil enzymes (neutrophil elastase, cathepsin G) degrade anti-tumorigenic TSP-1 - Brain-serine protease inhibitor (SERPINB1) determines brain tropism via ECM changes - Bone-cathepsin (e.g., cathepsin K), MMPs, and membrane-type MMPs mediate osteoclastogenesis and bone resorption	(40, 47, 54–62, 172)
PTMs (e.g., matrix crosslinking, glycosylation)	Physiological PTMs	Dysregulated glycosylation and collagen crosslinking	Collagen type I and type IV crosslinking by LOX family of enzymes (LOX, LOX-like 2 (LOXL2), and LOXL4) - Omentum-palmitic acid regulated LAMC1 posttranslational regulation	(13, 17, 30, 40, 41, 63–65)
Metabolites	Balanced and regulated	Altered glucose and lipid metabolism Increased release of GF, cytokines, etc.	- Lung, omentum-altered glucose (lactate production), and lipid metabolism (palmitic acid) promote ECM changes Increased release of GF, cytokines, etc.	(5, 30, 66, 67)
Other physicochemical parameters: hydration, pH, Ca^2+^ signaling	Hydrated and lubricated Tissue-dependent hydration Physiological pH Homeostatic Ca^2+^ signaling	Perturbations of ECM hydration Acidic pH Disrupted Ca^2+^ signaling	Decreased hydration due to altered GAG content and increased collagen crosslinking Disrupted levels of extra- and intracellular Ca^2+^ levels and dysregulated Ca^2+^ signaling delineated by increased production of Ca^2+^-binding proteins - Lung, LN: acidification of the ECM	(17, 35, 42, 66–69)

ECM, extracellular matrix; PGs, proteoglycans; GFs, growth factors; MMPs, matrix metalloproteinases; CAFs, cancer-associated fibroblasts; FNs, fibronectins; HA, hyaluronic acid; PMN, pre-metastatic niche; LN, lymph node; MDSCs, myeloid-derived suppressor cells; GAGs, glycosaminoglycans; BM, basement membrane; BBB, blood–brain barrier; EC, endothelial cell; BMDC, bone marrow-derived cell; EV, extracellular vesicle.

### Physiological ECM functions

The ECM stores and binds growth factors (GFs), cytokines, and extracellular vesicles (EVs), regulating their availability to cells depending on the physiological state of the tissue (5). The binding or trafficking of EVs, nanosized (50–150) membranous structures conveying bioactive cargo (proteins, lipids, metabolites, and nucleic acids) (78) throughout the ECM facilitates intercellular communication in the microenvironment (reviewed in 79, 80). A particular subtype of EVs, matrix-bound nanovesicles (MBVs), form an integral functional component of the connective tissue ECM by activating macrophages and supporting differentiation in neuroblastoma (81).

Communication between cells and their surrounding ECM occurs through focal adhesions and is mediated by ECM-binding receptors. Among these, integrins are the most prominent receptor family and are instrumental for the adhesion and migration of immune cells, thus facilitating systemic immune responses. Integrins bind ECM components such as fibronectin, laminin, collagen, and fibrinogen, as well as other integrins on the surface of cells (82–85). Non-integrin ECM receptors include glycoproteins such as syndecans, discoidin domain receptors, and CD44 (86–90). Importantly, by binding GFs and interacting with cell-surface receptors, the ECM elicits signal transduction and regulates gene transcription (1), thereby actively regulating cell growth, survival, differentiation, and migration. Matricellular proteins, such as TNC, TSP-1, and POSTN, alter the interactions between cellular transmembrane receptors and fibrous ECM molecules, thereby modulating cell proliferation and differentiation and playing a central role in tissue homeostasis (3). For example, hyaluronic acid (HA), also known as hyaluronan, is an anionic non-sulfated GAG synthesized by one of the three hyaluronan synthetases (HAS1, HAS2, and HAS3) (91) and a dominant constituent of the connective, epithelial, and neural ECM (32) essential for embryonic development, wound healing, and inflammation (91, 92).

### Cellular determinants of matrix deposition

Fibroblasts are the primary cellular components that “build” the connective tissue ECM and the major type of mechanoresponsive cells. In addition to being the primary source of most ECM components including collagens, PGs, elastin, growth factors [e.g., transforming growth factor beta (TGF-β) and platelet-derived growth factor (PDGF)] and cytokines [e.g., tumor necrosis factor-alpha (TNF-α) and interleukin 1 beta (IL-1β)], fibroblasts also secrete matrix metalloproteinases (MMPs), thereby emerging as the primary players in regulating tissue growth, ECM remodeling, wound healing, and fibrosis (22, 23). By producing both fibronectin and elastin, fibroblasts control the balance between stiffness and elasticity of the ECM, which in turn orchestrates cell migration, gene expression, and differentiation, as cells can sense ECM rigidity.

In cancer, quiescent and metabolically inactive fibroblasts are distinct from cancer-associated fibroblasts (CAFs), ubiquitous cells present in all solid tumors that become the major source of ECM molecules. CAFs develop from various cell types (e.g., stellate cells, bone marrow-derived cells (BMDCs), adipose tissue mesenchymal stromal cells (MSCs), and resident quiescent fibroblasts) and secrete higher levels of collagen I, FNs, laminin, and HA than normal fibroblasts, increasing ECM stiffness (24–26). FNs, laminin, and HA are also critical extracellular drivers of malignancy (6, 11, 63, 93) and reviewed in Frantz et al. (1) and LeBleu et al. (19).

### ECM alterations in cancer

**Figure 1:**
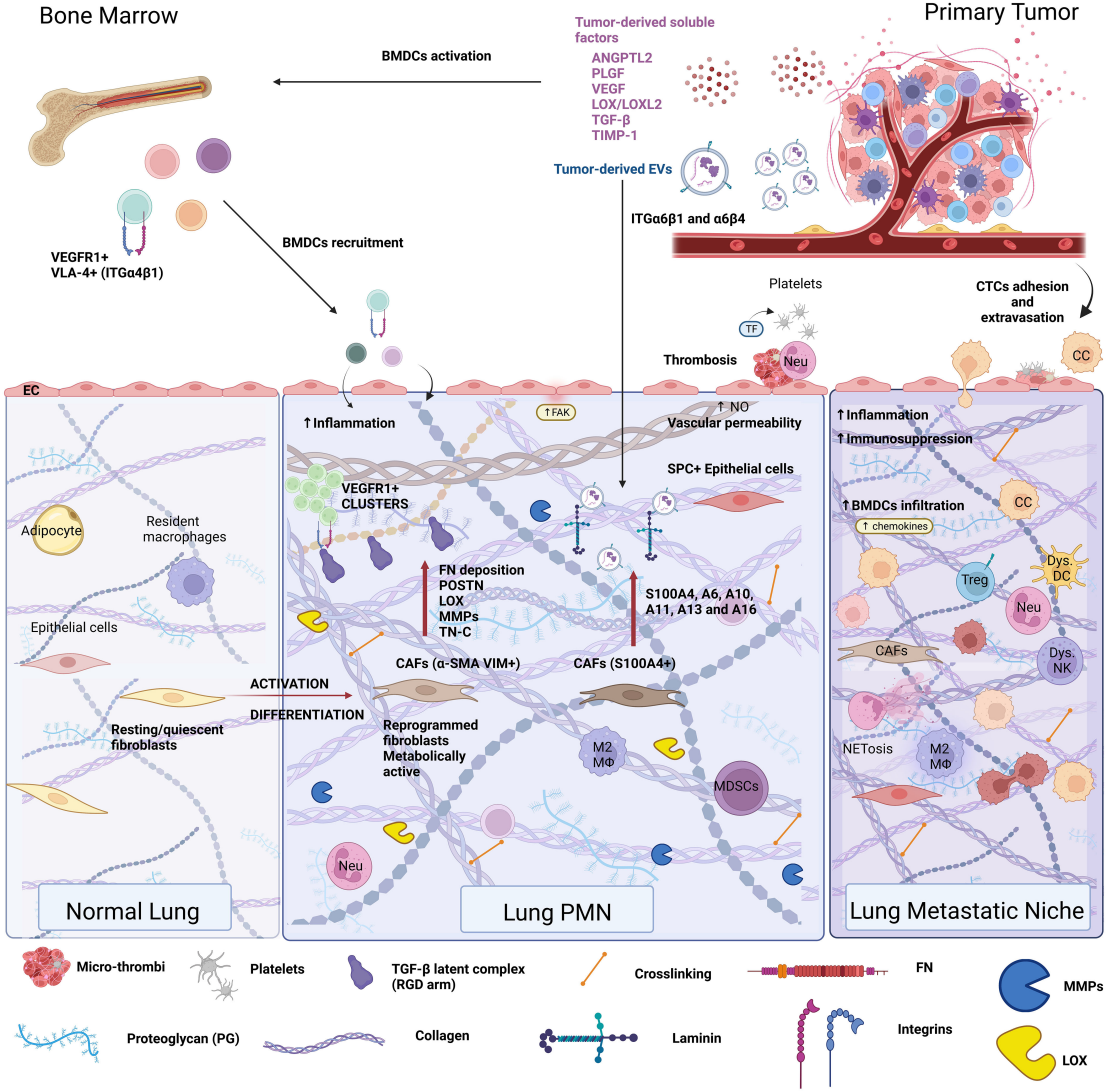
Extracellular matrix composition in the pre-metastatic niche. The remote conditioning of future organ sites of metastasis by the primary tumor is termed pre-metastatic niche formation. The lung pre-metastatic niche is depicted here, as it has been the most extensively characterized. Primary tumors secrete soluble factors, such as cytokines, chemokines, hormones, metabolites, and extracellular vesicles (EVs) that condition the future site of metastasis as well as the bone marrow. Bone marrow conditioning results in egress of VEGFR1^+^VLA-4^+^ bone marrow-derived progenitor cells, which are recruited to the pre-metastatic niche. Conditioning of the pre-metastatic niche induces vascular permeability, thrombosis, inflammation, and stromal cell activation, all of which cooperate in extracellular matrix remodeling. Pre-metastatic niche development generates a favorable milieu for circulating tumor cell (CTC) seeding and outgrowth, resulting in the formation of the metastatic niche.

Dysregulation of ECM homeostasis consists of alterations (e.g., ECM degradation and ECM deposition) in the dynamic ECM composition, organization, and mechanical properties that contribute to neoplastic progression and is a prerequisite for metastatic disease (3, 94). Typical changes in cancer-associated ECM were comprehensively reviewed by Frantz et al. (1). These include i) alterations in BM function with a loss of apical–basal polarity, along with cellular proliferation and metastatic cell migration; ii) contributions of differentiated fibroblasts (peritumoral fibroblasts, reactive stroma fibroblasts, CAFs, and myofibroblasts) to increased ECM deposition of collagen (types I, III, and IV), FNs, and elastin, as well as PGs (decorin, biglycan, lumican, and fibromodulin); iii) increased levels of TGF-β, VEGF, PDGF, EGF, bFGF, and HGF, promoting vessel growth and inflammation (1) (**Figure 1**).

Cancer-associated ECM alterations also include the loss of a differentiated phenotype. Interestingly, embryonic forms of FNs (i.e., oncofetal FNs) are re-expressed in cancer along with glycosylation dysregulation (95). Therefore, changes that perturb ECM homeostasis such as inflammation associated with wound healing, aging, obesity, or cancer include increased secretion and activation of enzymes that degrade the ECM (MMPs, serine, and threonine proteases), release stored GFs, and activate GF-mediated cellular functions. To this end, MMPs are essential in regulating the complex interplay between cells and the ECM (13).

Vascular BM components are also involved in the regulation of tumor angiogenesis (14, 74, 96). Matrix degradation promotes malignant progression and metastasis (97), as cancer cells strictly depend on ECM degradation for migration and intravasation and, thus, to escape their original environment. Moreover, the metastatic potential of most tumors correlates with the enzymatic degradation of BM collagen (98). Tumor microenvironment (TME) hypoxia drives ECM remodeling and collagen crosslinking to stiffen the tissue stroma during metastatic progression, promoting transformation, cancer cell survival, and outgrowth, motility, and invasion, ultimately enabling metastatic dissemination and facilitating the establishment of tumor cells at distant sites (40). Hypoxic tumors secrete LOX family enzymes that initiate ECM degradation and epithelial-to-mesenchymal transition (EMT), altering tumor cell behavior (40). Reducing LOX-mediated collagen crosslinking was sufficient to prevent tissue fibrosis and to increase ECM stiffness, focal adhesions, GF signaling, and cancer invasiveness in murine models of cancer (64). Changes associated with desmoplasia (i.e., growth of fibrous and connective tissue) are a common occurrence in tumors (e.g., pancreatic cancer (99)) and confer a fibrotic state due to increased ECM deposition and enhanced PTMs of ECM proteins (100). As a result, the tumor ECM has altered biomechanical properties due to crosslinking and orientation and has increased stiffness. Importantly, cancer cell interactions with a stiffer ECM provide them with a proliferative advantage (2) and instigate cancer progression (101).

Many matricellular proteins are increased in adult ECM when normal physiology is altered. Increased HA levels are observed in many tumor types and correlate with malignancy and metastasis (91, 102). HA specifically interacts with cell surface receptors CD44 and CD168 (also known as Receptor for Hyaluronan Mediated Motility) and can be retained on the cell surface after its synthesis. HA presence on the cell surface generates a voluminous pericellular matrix, which can further incorporate other HA-binding molecules; however, these interactions are not completely understood (91). In cancer, tumor cell–HA interactions influence intracellular signaling such as anti-apoptotic pathways and promote EMT and cell invasiveness (52, 91). POSTN also has a multifaceted role in tumor-associated ECM remodeling (103). In addition, tenascin C promotes glioma cell migration on fibronectin substrates by modulating interactions between αvβ5 integrin and FNs (3, 104). Tenascin C levels are elevated in the cancer-associated stroma, and they can inhibit the interaction between syndecan 4 and FNs, thus promoting tumor growth and metastasis (33).

Altogether, cancer-induced changes in tissue homeostasis alter ECM composition and physicochemical properties through ECM remodeling, ultimately leading to changes in ECM function.

### ECM regulation of angiogenesis—consequences for tumor progression

Of the ECM–stroma interactions, perhaps the most critical is the crosstalk with the vasculature, as ECM-mediated regulation of angiogenesis and vasculogenesis is essential for tumor growth, progression, and metastasis. Cancer neoangiogenesis is a complex process regulated through the intricate crosstalk between cancer cells, endothelial cells (ECs), and the ECM that dictates the potential of the tumor to metastasize (105). Since tumor growth is accompanied by hypoxia and limitations in nutrient availability, tumor vascularization, via angiogenesis and vasculogenesis, is a prerequisite for further tumor growth, escape of tumor cells from the primary site, and ultimately metastasis. Primary tumors depend on the VEGF-dependent recruitment of bone marrow-derived precursors (e.g., VEGFR1^+^ hematopoietic and VEGFR2^+^ circulating endothelial progenitor cells (EPCs) as a source of ECs) that initiate neovascularization (106) in response to specific TME ECM changes and the chemo-cytokine tumor- and tumor-associated stroma repertoire. For example, CAFs in the tumor stroma secrete stromal cell-derived factor (SDF-1)/CXCL12 and recruit CXCR4^+^ EPCs, thereby promoting new vessel formation (107). Importantly, the balance between positive and negative regulators of blood vessel formation directs a switch toward an angiogenic milieu in the TME (i.e., “angiogenic switch”), further supporting primary tumor growth and metastasis.

ECM-driven angiogenesis in cancer occurs through several mechanisms: i) the ECM provides a scaffold for EC migration and tube formation; ii) MMP-mediated ECM and BM degradation are required for tumor cell migration and as sources of pro-angiogenic growth factors (e.g., VEGF and FGF) (108); iii) ECM molecules such as FNs (109), HA (93, 108), TSP, angiostatin, and endostatin (73, 108, 110) directly regulate tumor angiogenesis. These mechanisms act in concert.

Matrix-degrading enzymes (MMPs), secreted by cancer cells or TME stroma (CAFs, adipocytes, and immune cells), are critical for neovascularization. MMP-1 secreted by breast cancer cells promotes angiogenesis and bone metastasis (111), while MMP-9 released by tumor-infiltrating neutrophils and macrophages triggers the angiogenic switch by releasing VEGF from tumor matrix to sustain angiogenesis-driven intravasation and metastasis (104, 112, 113). Moreover, vascular cell-derived MMP-9 induces pericyte detachment from vessels undergoing angiogenesis, destabilizing them and promoting vascular leakiness (114, 115). MMP-2, in turn, promotes angiogenesis by cleaving collagen IV and exposing a cryptic αVβ3 integrin binding site (55). MMP-2 and A disintegrin and metalloprotease 10 (ADAM10) also regulate vascular integrity and angiogenesis by degrading adhesion proteins at endothelial junctions, including VE-cadherin (56, 116). Interestingly, MMPs can also inhibit angiogenesis by generating endogenous angiogenesis inhibitors (e.g., cleavage of collagen chains and plasminogen) or by disrupting ligand–receptor interactions (117–120).

Judah Folkman pioneered the research into ECM components that inhibit angiogenesis and tumor growth, angiostatin, and endostatin (73, 110). Endostatin, a C-terminal cleavage product of collagen XVIII, a BM component, generated by MMP-3, MMP-7, MMP-9, MMP-13, MMP-20, or cathepsins (73, 118, 119, 121), interacts with ECs via integrins, glypicans, or VEGFR-2 on their surface (122), inhibiting VEGF and bFGF-induced EC migration and inducing apoptosis (123). Angiostatin, the most potent anti-angiogenic factor, results from proteolytic cleavage by extracellular urokinase, plasminogen activators, MMP-3, MMP-7, MMP-9, and MMP-12 (117, 124–126), and is systemically released by primary tumors, suppressing primary tumor growth and metastasis, explaining why primary tumor removal can lead to outgrowth of occult, pre-existing micro-metastases (73, 127).

Another endogenous matricellular inhibitor of angiogenesis is TSP-1, a calcium-binding glycoprotein belonging to the thrombospondin family that exerts its anti-tumorigenic activity on both tumor and endothelial cells (128, 129), reducing VEGF expression (130, 131) and disrupting VEGF signaling (132). However, TSP-1 also mediates the invasiveness and migratory capacity of several cancers such as melanoma, thyroid, and prostate (133–136) by binding both ECM (FNs, fibrinogen, laminin, and collagen IV) as well as integrins. Indeed, loss of TSP-1 is regarded as an “angiogenic switch” that could drive tumor progression (137). Thus, the TSP-1 role in mediating ECM-dependent tumor progression and angiogenesis is likely highly context and microenvironment dependent.

Integrins, receptors for ECM such as collagen on smooth muscle (α1β1) or epithelial cells and platelets (α2β1), also modulate angiogenesis. For example, integrin α1β1, the major collagen IV receptor, activates endothelial cell signaling pathways leading to proliferation, adhesion, migration, and tubulogenesis (138), while also promoting fibroblast proliferation and inhibiting the synthesis of collagen and MMPs. Integrin α1-deficiency markedly decreased tumor vascularization and increased angiostatin levels in plasma via MMP-7 and MMP-9 (139). FNs, the α5β1 integrin ligand on ECs, are also involved in promoting angiogenesis in a VEGF-independent manner and blocking α5β1 integrin with antibodies, peptides, or antagonists promoting tumor regression (109).

Integrin-mediated signaling is augmented by HA, which is highly produced by tumor or stromal cells (102, 140). HA critically regulates endothelial barrier function, and its processing profoundly impacts cancer-associated vascularization (141, 142). Moreover, HA stimulates EC tube formation via CD44 and CD168/TGFβ Receptor I signaling, which promote plasminogen activator-inhibitor-1 (PAI-1) expression and subsequently ICAM-1, VCAM-1, and MMP-2 expression, thereby promoting tumor angiogenesis (93, 143, 144).

Taken together, these findings support the idea that tumor-associated ECM alterations provide pivotal biochemical and biomechanical cues to cancer and stromal cells in the tumor microenvironment, emerging as critical modulators of cancer hallmarks and virtually supporting every malignant characteristic of tumor progression, such as sustained proliferation, growth, angiogenesis and evasion from apoptosis or antitumor immunity, chronic inflammation, dysregulation of cellular energetics, and acquisition of invasiveness potential (2).

## ECM changes as a hallmark of the PMN

### ECM composition in the PMN

Ever since the discovery of the pre-metastatic niche (PMN) (40, 48, 145), ECM changes have been recognized as a hallmark of PMN formation. ECM remodeling is a prerequisite for PMN initiation by preconditioning the niche, “weaving the nest”, to create a favorable environment for metastatic cell seeding (146, 147). The primary tumor induces ECM deposition at distant sites through its secretome (soluble factors such as cytokines, chemokines, growth factors, and EVs) with angiogenic, inflammatory, chemoattractant, and enzymatic roles that initiate PMN formation (**Table 1** and **Figure 1**).

ECM changes at the pre-metastatic site resemble alterations associated with aging, or primary tumor progression, such as increased fibronectin secretion (27, 48, 148) and stiffness and result from activation and differentiation of stromal cell phenotypes. Specifically, senescent fibroblasts in aging, or pro-tumor fibroblast differentiation in cancer, deposit more fibronectin. PMN-specific changes consist of ECM deposition (mainly mediated by resident-activated stroma/immune cells or infiltrating immune cells) and ECM degradation that altogether remodel the architecture of the distant tissue microenvironment (**Figure 1**).

Upregulation of FNs and increased FN deposition by activated resident fibroblasts in the lung and resident macrophages in the liver PMN have been described in murine and human models of melanoma (34, 48) and pancreatic cancer (27, 148), as well as in uninvolved lymph nodes of breast cancer patients (149). Fibronectin-rich pre-metastatic organs (lung, liver, testis, spleen, and kidney) are hospitable niches for the recruitment and adhesion of VEGFR1^+^VLA-4^+^ BMDCs in murine melanoma and lung cancer models and also in human breast and gastrointestinal cancers, highlighting the clinical relevance of this finding (48). Pancreatic CAFs (α-SMA^+^) support liver ECM remodeling via pro-inflammatory IL-6 secretion and induction of IL-6/JAK1/STAT3 signaling in hepatocytes that in turn promote the production of FNs and collagen I in the liver (65).

Another matricellular protein known for its various pro-tumorigenic roles in solid cancers (150) and with an emerging role in pre-conditioning the PMN is POSTN. For example, primary tumor-derived TGF-β induces the expression and secretion of POSTN by stromal fibroblasts (α-SMA^+^VIM^+^) in the pre-metastatic lung of the MMTV-PyMT breast cancer mouse model, sustaining infiltrating metastasis-initiating cells by promoting WNT signaling (151). POSTN also interacts with type I collagen, fibronectin, NOTCH1, TNC, and BMP1 and engages with αvβ3 and αvβ5 integrins, promoting cell motility throughout the tissue parenchyma (152). Importantly, POSTN is involved in tissue repair and upregulated in damaged tissues, where it also contributes to creating a PMN-like microenvironment during tissue regeneration (152).

Among PGs, versican (VCAN) is key to ECM remodeling at the distant future sites of metastasis, with various functions depending on the cellular source (49, 153). For example, in lung cancer PMN, tumor-derived VCAN activated macrophages, generating an inflammatory environment (153), whereas, in the MMTV-PyMT model, the BMDC-derived VCAN was pivotal for lung PMN development (49).

### ECM-mediated regulation of angiogenesis in the PMN

Primary tumor angiogenesis is closely related to PMN formation, as it entails the chronic release of PMN-inducing factors, such as VEGF, SDF-1, TGF-β, and TNF-α (154), that ultimately reconfigure future metastatic sites to create a permissive niche for cancer cell extravasation and proliferation. Primary tumor-secreted factors, such as VEGF, then induce MMP-9 in lung ECs and macrophages (145). The primary tumor secretome also remotely mobilizes BMDCs to the early PMN through mechanisms that rely on interdependent vascular and ECM remodeling. The seminal paper describing this effect showed that VEGFR1^+^ hematopoietic progenitor cells (HPCs) are anchored in FN-rich areas of the lung via integrin α4β1 (VLA-4) (48) initiating a feed-forward loop that amplifies ECM remodeling and compromises vascular integrity (e.g., FNs, LOX, and S100A8) (155–157). Also recruited to the lung PMN are CD11b^+^Gr1^+^ myeloid progenitor cells, which are major contributors to VCAN (an ECM proteoglycan) deposition in the pre- and metastatic lungs (49). VCAN then promotes mesenchymal-to-epithelial transition (MET) of metastatic tumor cells by interfering with Smad2 activation downstream of TGF-β signaling, leading to metastatic outgrowth (49).

Importantly, circulating anti-angiogenic factors such as TSP-1, angiostatin, and endostatin can counteract the effects of pro-angiogenic tumor-secreted factors, thereby preventing PMN formation. However, TSP-1 can also exert pro-metastatic effects as evidenced in breast cancer cells *in vitro* (158) and in osteosarcoma patients, in which both lung metastatic primary tumors and lung metastases expressed higher levels of TSP-1 in response to TGF-β, inducing further ECM remodeling by promoting MMP-2, MMP-9, and FN1 (159). However, the pro-metastatic functions of TSP-1 are overshadowed by its anti-metastatic roles (57, 160). For example, in response to tumor-secreted prosaposin (PSAP), Gr1^+^ BMDC secretes TSP-1 at the PMN site, hindering metastasis, and PSAP mimetic peptides could recapitulate this effect (160). The effects in the distant organ could be ascribed to TSP-1 anti-angiogenic function (130, 131) and blocking of the migration and recruitment of VEGFR1^+^, VEGF-responsive BMDCs critical for PMN initiation (48, 106, 161). Future studies may unveil the potential of the systemic PSAP/TSP-1 axis in modulating PMN formation (161) and thus could provide insight into the role of circulating anti-angiogenic molecules and circulating ECM in systemic regulation of the “pre-metastatic switch”.

### ECM degradation and remodeling at the PMN

ECM degradation and remodeling are achieved via enzymatic breakage of the ECM by proteases, such as MMPs, serine, and threonine proteases, to generate bioactive molecules as well as reconfigure collagen fibers via enzymatic crosslinking by LOX family enzymes (40, 145, 162). Enzymatic modulation of the PMN ECM is also intricately linked to vascular integrity, as vascular leakiness is another hallmark of PMN formation (163). Major sources of ECM remodeling MMPs are BMDCs (macrophages, CD11b+ myeloid cells, and VEGFR1^+^ HPCs), stromal fibroblasts, and endothelial cells (40, 48, 145, 147, 164). Importantly, collagen IV peptides resulting from MMP-2 and MMP-9 mediated cleavage chemoattract BMDCs and circulating tumor cells (CTCs) to PMNs and promote metastasis (40). Another ECM remodeling molecule, TIMP-1, specifically cleaves MMPs and controls their enzymatic activities locally (162). High levels of TIMP-1 enhance cancer cell propensity for liver metastasis in murine and human pancreatic, lung, gastric, and ovarian cancers, lymphoma, and fibrosarcoma models (165). In osteosarcoma models, tumor-secreted pro-inflammatory glycoprotein angiopoietin-like protein 2 (ANGPTL2) binding to integrin α5β1 mediates tumor cell intravasation via MMPs, enhancing lung metastasis (166).

LOX family enzymes are critical modulators of PMN ECM remodeling in many cancers, particularly breast cancer. Hypoxic tumors, along with HIF1 activation (e.g., breast cancer and head and neck cancer) are the main source of LOX, which is released systemically and disrupts ECM homeostasis in the pre-metastatic lung or bone (167) by crosslinking ECM molecules and primarily collagen. Collagen type I and type IV crosslinking by LOX, LOX-like 2 (LOXL2), and LOXL4 that accumulate in PMNs of various organs create an adhesion platform for CD11b+ BMDCs (40), which further contributes to PMN formation through a positive feedback loop of MMP production that facilitates the colonization of the distant tissue by CTCs (40, 58, 168). In addition to tumor cells, activated fibroblasts also represent a source of LOX in the pre-metastatic tissue (40, 58).

### Extracellular vesicles shape the pre-metastatic niche ECM

Small EVs or exosomes are nanosized (50–150) membranous structures released by cells into the extracellular space and systemically mediate intercellular communication, modulating the behavior of the target cells and their microenvironment (169). Tumor-derived EVs exert systemic effects and convey malignant traits from primary tumors to distant organs via functional interactions and/or specific transfer of cargo [proteins, lipids, metabolites, and RNA such as microRNA (miRNA)] that contributes to organotropic metastasis, ECM remodeling, and reprogramming resident cells in the distant tissue (e.g., Kupffer cells, microglia, fibroblasts, and ECs). EVs promote ECM remodeling by altering the physiological architecture of the niche (27, 28, 35, 170, 171). Several studies highlighted the role of primary tumor-derived EVs in promoting ECM remodeling in the pre-metastatic lungs in melanoma (34), pancreatic cancer (41, 59), osteosarcoma (170), nasopharyngeal carcinoma (171), metastatic adenocarcinoma (172), liver (27), pancreatic (27) and gastric (173) cancer models and lymph nodes (LNs) in melanoma (174) and metastatic adenocarcinoma (172) models.

## Physicochemical properties of the pre-metastatic ECM

The main physicochemical alterations of the PMN ECM, involving changes in tension, pH, ion composition, and hydration, are driven by BM dysfunction, vascular leakiness, thrombus formation, and changes in the ECM composition such as increased production of ECM proteins and their deposition and increased production of ECM-degrading enzymes that disrupt ECM physiology. Thus, physicochemical ECM changes contribute to the fertile “soil” of the PMN tissue, characterized by a pro-invasive ECM. Specifically, the major physicochemical changes of the ECM encompass changes that impart mechanical properties (ECM mechanics) in relation to molecular weight, size of the ECM, and assembly (structural), and chemical changes such as ionization state, pH, and hydration, which have not been yet studied extensively in the context of the PMN.

Increased fibronectin secretion and deposition (assembling into fibronectin fibrils) (27, 29, 48, 148) increase collagen production and subsequent crosslinking by LOX enzymes (40, 65), imparting major structural changes on the ECM characteristic of the liver and lung PMNs. The pathological matrix deposition and crosslinking disrupt ECM mechano-homeostasis by increasing stiffness (i.e., mechanical force and tensile strength), reducing elasticity and the swelling ratio, characteristic for a fibrotic environment that is biomechanically less compliant and more rigid (175; 29). Therefore, the increase in mechanical force further facilitates BMDC infiltration, creating a highly inflammatory environment (40), and a dense matrix that prevents T-cell recruitment and function, as reported in tumors (176). Concomitantly, the crosslinking of collagen is associated with fiber dehydration and a fibrotic ECM (42). Thus, future studies assessing the PMN hydration state, along with the proteoglycan dysregulation, are warranted.

The inflammation occurring during early PMN formation is accompanied by higher ionization, as described by Kurpińska et al., who showed that calcium (Ca^2+^) signaling was dominant in the lung PMN as a pre-requisite for cytoskeleton and ECM reorganization (66). Among the most prominent mediators of the PMN inflammatory circuitry are the S100 proteins (e.g., S100A4, S100A9, and S100A8/A9), small Ca^2+^ signaling molecules that are hallmarks of lung and liver PMN formation (35, 68, 69, 177). The S100 family proteins bind to many proteins including the receptor for advanced glycation end products (RAGE) and extracellular matrix metalloproteinase inducer (EMMPRIN) (69, 178, 179). These receptors are known to promote ECM deposition and enhance pro-invasive ECM properties via MMP induction. Importantly, extracellular Ca^2+^ can regulate post-translational MMP dynamics (180), thereby showing that the activity of Zn^2+^-dependent MMP endopeptidases depends not only on the intracellular Zn^2+^ but also on increased Ca^2+^ availability and inflammation. Other Ca^2+^-dependent interactions with ECM molecules include the binding of endostatin to transglutaminase 2 (122) and the dependence of ADAM10-mediated proteolysis on Ca^2+^ influx (56).

Interestingly, in breast cancer, extravesicular CEMIP promoted brain PMN formation (28) potentially by depolymerizing HA (181), as well as intracellular Ca^2+^ regulation by releasing Ca^2+^ from the endoplasmic reticulum deposits (182). Therefore, intracellular calcium release in brain ECs may mediate calcium-dependent cytoskeletal re-arrangements and EC migration (28, 183). Collectively, these studies suggest that disruption of Ca^2+^ homeostasis may be critical for pre-metastatic ECM reorganization and PMN formation.

Although no exhaustive studies reported data on pH levels in the PMN, several findings suggest that lung PMN could foster an acidic microenvironment. Interstitial macrophages in the lung metabolically reprogrammed by tumor-derived EVs displayed a glycolytic-dominant metabolic profile, which fueled the conversion of pyruvate to lactate and lactate secretion, thereby contributing to the extracellular milieu acidification (67). Moreover, early lung PMN changes in a breast cancer model were associated with altered glucose metabolism and a decrease in lactate dehydrogenase chain b (Ldhb) gene expression (66), which catalyzes the conversion of lactate and NAD^+^, to pyruvate with and NADH and H^+^, ultimately contributing to intracellular or extracellular acidification depending on monocarboxylate transporter expression (184). Tumor-derived lactic acid activated the fibroblastic reticular cells in the draining LNs, indicated by increased alpha-SMA, and induced collagen I production along with increased expression of genes involved in ECM remodeling. These changes supported the formation of a pro-fibrotic environment favorable for PMN formation (43). Thus, PMN acidification may play a pro-metastatic role.

In the context of cancer, CAF-secreted EVs mediated a decrease of intracellular lactate in breast cancer cells with a concomitant extracellular environment acidification (185). Whether PMN CAF-derived exosomes can exert similar effects locally remains to be determined. However, it is important to stress that a complete metabolic landscape of the PMN has not yet been established. Thus, it would be important to explore the relationship between lactate production, pH level increases in the PMN and ECM remodeling, and the mechanisms through which they cooperate to promote metastasis.

## Organ-specific ECM remodeling and functional outcome

Several seminal studies (48, 145) demonstrated the propensity of cancer cells to metastasize to specific organs—defined as organotropism—reinforcing Paget’s “seed and soil” theory (186). In this review, we explore the role of the ECM in orchestrating organotropic metastases and emphasize the importance of the “soil” and the specific local interactions that render the soil amenable for successful metastatic seeding. Tumor-derived small EVs orchestrate tumor metastasis to specific organs with a prominent role ascribed to integrins that dictate specific interactions with target cells (27, 28, 35). Along with ECM structure garnering greater interest in cancer metastasis, several studies demonstrated that EVs actively contribute to ECM remodeling at distant sites by reprogramming stromal cells (e.g., fibroblasts, epithelial cells, resident macrophages, and recruited immune cells) to alter the architecture of the microenvironment (27, 35, 41, 170, 171).

### PMN-associated ECM remodeling in the lung

The lung was the first metastatic site where pioneering PMN research demonstrated that MMP-9 expression in endothelial cells and macrophages was induced by melanoma-secreted VEGF (145) and that FN (α4β1 ligand) was upregulated by VEGFR1^+^ integrin α4β1^+^ (VLA-4^+^) bone marrow-derived HPCs mobilized to the lung via tumor-secreted VEGF-A and placental growth factor (PIGF) (48) (**Figure 1**). In breast cancer, essential ECM components dictate the propensity for lung metastases at both cellular and extravesicular levels. Expression of integrin α3β1 on murine mammary carcinoma (4T1) cells was associated with lung colonization via binding laminin α5 (LAMA5) (187), which is widely expressed on the endothelial cell BM in lung capillaries (188). Human lung-tropic breast cancer-derived EVs package laminin-binding ITGα6β4 and ITGα6β1 (35). Moreover, specific uptake of these EVs by S100A4+ fibroblasts in laminin-rich lung microenvironments activated Src and pro-inflammatory S100 gene expression in these cells (35).

Through their pleiotropic functions and prominent role in ECM deposition and remodeling, lung fibroblasts are instrumental players in pre-conditioning the distant organ to create a permissive environment for metastatic colonization by shaping the PMN immune microenvironment (34, 40, 48, 151). Early changes induced at the distant site by the primary tumor include the differentiation of fibroblasts, which are sensitive to ECM mechanics (44). Importantly, lung fibroblasts are also a common target for tumor-derived EVs, with a large number of studies demonstrating that EV-specific functional cargo could induce fibroblast activation and differentiation into myofibroblast or CAF phenotypes, promoting ECM remodeling at the pre-metastatic site (35, 41, 170, 171).

Reprogrammed fibroblasts deposit FN, which becomes an adhesion platform for incoming BMDCs via their integrins (48, 82, 147, 148) and secretes POSTN, which promotes myeloid-derived suppressor cell (MDSC) accumulation and an immune-suppressive environment (189), and CTC colonization (151). The primary tumor-derived or local production of LOX enzymes (LOX and LOXL2) along with the increase in FNs and collagen creates a profibrotic lung microenvironment that is conducive to PMN formation and further supports inflammation and immunosuppression (40, 58, 63, 190). Lastly, a recent study showed that cytotoxic treatment of primary breast cancer (e.g., doxorubicin) induced the functional differentiation of lung PMN-promoting CAFs toward a pro-inflammatory phenotype, albeit with reduced cell migration and contractility and downregulation of fibrosis-related gene signature (191). These results support the idea that therapeutic intervention aimed at re-educating CAFs could hinder ECM remodeling and subsequent PMN formation.

ECM remodeling is intricately linked to vascular permeability and thrombosis, established hallmarks of PMN formation. Due to its structural features, lung tissue enhances the propensity of circulating tumor cells arrest in the dense capillaries and extravasate into the lung parenchyma (192). Cancer also induces systemic thrombosis, which consists of the adhesion and aggregation of platelets (163). Thrombosis is a bona fide example of cell–matrix interactions that occur when vascular integrity is compromised, exposing the BM and IM. Platelet-derived thrombin converts fibrinogen to fibrin that further crosslinks to fibronectin, resulting in platelet aggregation and thrombus formation. Fibronectin deposited at PMNs consequently acts as a scaffold for microthrombi. Fibrin and its precursor fibrinogen are ECM components that drive thrombosis at the distant site. Fibrinogen release from blood vessels and its deposition as fibrin in the pre-metastatic lung of murine melanoma models alters the vasculature integrity and favors microthrombus formation (163). Additionally, platelet ADP receptor P2Y12 activation by ITGαIIbβ3 [a ligand for RGD and AGD-containing ligands, such as fibrinogen, fibrin, and von Willebrand factor (83)] induced platelet aggregation in the lung PMN, promoting VEGFR1^+^ BMDC recruitment and increasing fibronectin deposition (193).

Innate immune cells that infiltrate the lung and participate in PMN formation, namely, neutrophils, have been shown to promote the release of elastase and cathepsin G proteases, leading to the degradation of TSP-1, which in turn enhanced lung metastasis. Neutrophils contribute to the proteolytic degradation of anti-tumorigenic TSP-1 via neutrophil-mediated degranulation of azurophilic granules to release serine proteases, such as neutrophil elastase (NE) and cathepsin G (57). Thus, the upregulation of neutrophil proteolytic activity and subsequent loss of TSP-1 underlie the PMN lung inflammatory microenvironment and metastatic outgrowth (57). Moreover, neutrophil extracellular trap (NET) formation activated tumor growth by degrading TSP-1 (57, 60), and inflammation-activated neutrophils proteolytically disrupt TSP-1 to enable cancer cell colonization (60) by an unknown mechanism.

Neutrophils play an undisputed critical role in cancer metastasis and their pro-metastatic role as well as functional effects in the PMNs, which is drawing increasing attention. Primary tumor-secreted Cathepsin C (CTSC), a protease that activates other proteases driving the proteolytic remodeling of the ECM, regulates neutrophil infiltration and NET formation *in vivo*, being critical for the early stage of metastatic colonization of the lung microenvironment by breast cancer cells (60). CTSC levels are highly increased in metastases compared to the primary tumors of breast cancer patients (60). Under inflammatory conditions, the proteolytic ECM remodeling activity of NETs awakened cancer cells from dormancy (194), while NET-associated DNA induced cancer cell chemotaxis (195).

Interestingly, a recent study showed that systemic communication and cooperation with a third organ were involved in regulating the development of PMNs and metastases. For example, liver-entrained leukocytes (B220^+^CD11b^+^NK1.1^+^) expressing coagulation factor X (FX) and vitronectin (VTN)—an adhesive glycoprotein that connects cells and ECM via integrins—traveled to fibrinogen‐rich hyperpermeable regions in pre‐metastatic lungs, where upon induction of TSP expression, they eliminated fibrinogen deposition, thus interfering with thrombotic and fibrotic events in the lung (196).

### EV-mediated pre-conditioning of the ECM in the lung PMN

A variety of EV cargo modulates ECM deposition and function in the PMN. Melanoma EVs conveying onco-fetal insulin-like growth factor 2 mRNA-binding protein 1, involved in post-transcriptional regulation of transcripts encoding proteins for cell adhesion, migration, and ECM remodeling [e.g., CD44, a ligand for HA, osteopontin (OPN), collagens, and MMPs (168)], promoted ECM remodeling and FN deposition (34).

EVs secreted by murine primary pancreatic adenocarcinomas generate a pro-invasive lung niche by evoking invasive alterations to ECM deposition and architecture. Mechanistically, controlled EV loading of podocalyxin—a glycocalyx component and adhesion receptor with negative charge imparted by glycosylation—influenced Rab-coupling protein-dependent ITGα5β1 trafficking in normal fibroblasts in the pre-metastatic lung and altered ECM architecture (i.e., increased orthogonal characteristics), rendering the microenvironment conducive for metastatic cell seeding (41). EV-packaged latent membrane protein 1 (LMP-1) also induced lung fibroblast differentiation into CAFs (expressing α-SMA and fibroblast activation protein (FAP)) *in vivo* in a nasopharyngeal carcinoma model (171). Meanwhile, osteosarcoma-derived EVs enriched in TGFβ1 activated lung myofibroblast/CAFs as demonstrated by increased α-SMA expression and FN production (170). Moreover, EVs enriched in TNF1α, TGFβ, and IL-6, isolated from hypoxic human prostate (PC3) cancer cells, increased MMP-2, MMP-9, fibronectin, and collagen deposition and stimulated the recruitment of CD11b myeloid cells at the PMN (59). Lastly, EVs from metastatic rat adenocarcinoma (ASML) pre-conditioned the lung for metastasis via CD44v-dependent transcriptional and posttranscriptional regulation of miRNA repertoires. Some of these miRNAs, such as miR-494 and miR-542-3p, target cadherin-17 and can upregulate MMP transcription *in vitro*, thereby promoting ECM remodeling via functionally modulating target stroma cells in the lung and LNs (172).

### PMN-associated ECM remodeling in the liver

Pancreatic cancer-derived EVs enriched in RGD-motif binding integrin αvβ5 exert liver tropism and specifically bind to Kupffer cells, liver-resident macrophages (F4/80^+^) with phagocytic function, reprogramming them through Src activation and the expression of pro-inflammatory S100 gene expression (35). Kupffer cells are intrinsically susceptible to EV-mediated reprogramming and can modulate ECM changes associated with PMN formation and induction of a pro-metastatic inflammatory milieu that supports tumor colonization. Pancreatic cancer-derived EVs enriched in macrophage inhibitory factor (MIF) were internalized by Kupffer cells (F4/80^+^) activating signaling pathways (e.g., TGF-β secretion) that reprogrammed hepatic stellate cells (HStCs) (α-SMA^+^desmin^+^) toward a myofibroblastic phenotype with increased FN production and deposition that creates a fibrotic environment. ECM remodeling further stimulated BMDC myeloid and neutrophil recruitment to the liver, facilitating PMN formation (27). Another study recently showed that extravesicular ANGPTL1 suppressed MMP-9 production in Kupffer cells, hindering liver vascular leakiness in colorectal cancer (CRC) murine models (197). However, the exosomal miR-135a-5p released from hypoxic colon cancer cells promoted MMP-7 production in Kupffer cells (198).

In CRC-associated liver metastasis, CCL15 mobilized immature myeloid cells to the pre-metastatic liver, producing MMP-2 and MMP-9 that degraded the liver ECM (199). EV-packaged CCL2 induced M2 pro-tumor macrophage differentiation and liver fibrosis that rendered the liver environment more conducive for cancer cell invasion (29). Another ECM modeling molecule, TIMP1, packaged into CRC EVs induced a positive feedback loop by upregulating TIMP1 in recipient fibroblasts in the liver, also resulting in ECM remodeling, as shown in human HCT116, HT29, and SW620 CRC models (44). Moreover, EV-mediated transfer of miR-122-5 upregulated N-cadherin and Vimentin and downregulated E-cadherin in hepatocytes in non-small cell lung cancer (NSCLC), thereby regulating PMN-promoting cell–ECM adhesions (36). Finally, gastric cancer-derived EV EGFR regulated the propensity of gastric cancer cells to colonize the liver by educating stromal cells in the microenvironment. Thus, EV-mediated translocation of EGFR to the plasma membrane of liver stromal cells activated HGF/c-Met signaling, facilitating gastric cancer cell invasion and liver seeding (173).

Altogether, these studies demonstrate that soluble factors or EVs released by primary tumors establish a fibrotic PMN-promoting liver environment via reprogramming local stroma or immune cells (e.g., Kupffer cells and HStCs) and highlight the therapeutic potential of targeting circulating EVs and/or the fibrotic niche for early prevention and therapeutic intervention against cancers with a propensity for liver metastasis.

### PMN-associated ECM remodeling in the brain

The brain ECM has unique features: the most abundant ECM component is HA, while collagen is less prevalent, and FN is concentrated around the BM (37), as reviewed in Deasy and Erez (38).

Integrin β3 expression promotes brain tropism and brain PMN formation (200). Another molecule that shows specificity for brain metastatic tropism is a serine protease inhibitor, SERPINB1, highlighting the fact that ECM changes occur in the brain PMN (61). The main cellular players in promoting brain PMN formation are microglia and brain-resident macrophages. Microglia specifically uptake EVs released by brain-tropic metastatic cells, promoting neuroinflammation (i.e., perivascular inflammation) and increased cell adhesion, diapedesis, in the brain microenvironment due to the specific enrichment of hyaluronan-binding protein, also known as hyaluronidase 1 (CEMIP) in these vesicles (28). CEMIP breaks HA, and the resulting by-products are known activators of angiogenesis (32), consistent with the local vascular remodeling (i.e., disruption of the blood–brain barrier) in the pre-metastatic brain (28, 35). Thus, endothelial vasculature is susceptible to alterations mediated by tumor-derived EVs. For example, breast cancer EV-mediated transfer of miR-181c to brain endothelial cells disrupts the blood–brain barrier (45), whereas glioblastoma stem-like cell-derived EV transfer of VEGF-A to endothelial cells stimulated angiogenesis (201).

Interestingly, pro-metastatic EVs secreted by aggressive brain-tropic breast cancer models (MDA-MB-231-BrM2-831) interact systemically with low-density lipoprotein (LDL), which increases their propensity to be internalized by monocytes in the brain microenvironment (202). In contrast, their junctional interactions (e.g., including zona occludens 1, N-cadherin, VE-cadherin, claudin-5, and occludin) with human brain microvascular endothelial cells were similar to those of EVs isolated from non-tropic breast cancer (MDA-MB-231) (202). This study highlights the fact that systemic interactions with other circulating factors synergize with EV-intrinsic properties to determine their functional properties.

### PMN-associated ECM remodeling in the bone

Bone has a unique collagen makeup, providing this tissue with increased strength through the mineralization of type I collagen and contains areas with dense or porous ECM matrix (203). The bone marrow is a complex microenvironment that contains multiple cell types of bone stromal cells that contribute to ECM remodeling in the PMN; osteoblasts, myeloid lineage osteoclasts, and fibroblasts are prominent. However, osteoblasts are the specialized cells primarily responsible for bone ECM matrix production.

Various ECM interactions with cellular or extravesicular integrins determine bone tropic metastasis. For example, human and murine breast cancer-secreted EVs enriched in cadherin 11 (CDH11) and α5 integrin delivered runt-related transcription factor 2 (RUNX2) to the bone, inducing an osteogenic environment and bone lesions that promoted bone colonization by cancer cells (46). Increased α5 integrin in breast cancers or bone was associated with osteoclast-mediated bone resorption and osteolytic metastases (50a). Additional integrins on the surface of prostate and breast cancer cells that mediate bone niche colonization are integrins β1 and β3 (51, 53). Integrin interactions and signaling are also pivotal for pancreatic cancer cell migration to the bone. Specifically, pancreatic cancer cell adhesion to collagen I is mediated via ITGα2β1 and stimulates their migration and subsequent bone colonization (51), whereas integrin-mediated cancer–stroma interactions in the bone have various outcomes: the expression of αIIbβ3 promotes platelet aggregation, similarly to the lung, and integrin signaling via αvβ3 and VLA-4 on tumor cells promotes tumor metastasis in the bone niche (53).

Among the cell types shaping the bone ECM, circulating platelets also play an important role in bone remodeling by mediating the crosstalk between the tumor and the bone microenvironment. Sequestration of functional molecules by platelets (platelet TSP-1, TGF-β1, MMP-1, and receptor activator of NF-κB (RANK)) mediates bone-tropic prostate cancer-induced osteoclast differentiation and BMDC infiltration (62, 204). Similarly, the receptor activator of NF-κB ligand (RANKL) regulated osteoclastogenesis and induced bone remodeling and PMN formation (205). Fibroblasts or fibroblast-like cells directly enhance bone resorption by inducing osteoclastogenesis by producing cathepsin (e.g., cathepsin K), MMPs, and MT MMPs (47). However, the crosstalk between these stromal cells in PMN ECM remodeling is not completely understood.

### Lymph node PMN-associated ECM

The majority of cancers involve LN metastasis, which is an important clinical indicator of patient outcome (149, 174, 206, 207). LNs are also susceptible to reprogramming or remodeling mediated by EVs, as lymphatics are a major trafficking route for tumor-derived EVs. A recent study described that EV integrin mediated homing to LN in murine melanoma, as well as their target in the pre-metastatic tissue. Specifically, tumor-derived EVs enriched in α4β1 (VLA‐4) or α9β1 were internalized only by lymphatic endothelial cells (VCAM-1^+^) from the subcapsular and medullary sinuses of B16-F10 melanoma-bearing mouse draining lymph nodes. This resulted in EV-shuttled antigens that mediated immune escape and killing of cytotoxic CD8+ T cells (207). Melanoma EV homed to sentinel LNs and promoted LN remodeling into a hospitable niche before tumor cell colonization by inducing the expression of factors responsible for cell recruitment (GM-CSF in endothelial cells), ECM remodeling, and angiogenesis (174).

In the metastatic rat adenocarcinoma model BSp73ASML (ASML), which has increased susceptibility for PMN formation in LNs and lung tissue, exosomal miR-494 and miR-542-3p transfer to lymph node stromal cells and lung fibroblasts, resulting in the transcriptional upregulation of MMPs and downregulation of cadherin-17, thereby mainly affecting proteases and adhesion molecules, highlighting the myriad of mechanisms through which EVs mediate ECM changes in pre-metastatic tissues (172).

### Peritoneal and omental PMN-associated ECM

The omentum, a common site for ovarian metastasis, has received great interest in the past years due to its aggressive nature and late diagnosis of ovarian cancer. A recent study revealed collagen-rich omentum as a homing site for ITGα2-mediated peritoneal metastases (39). Omental ECM proteins contributed to early metastasis to the peritoneum by triggering cancer cell adhesion to collagen, cell migration, and activation of focal adhesion kinase. Importantly, both ITGα2 and altered collagen expression in the pre-metastatic omentum were indicative of poor clinical outcomes in ovarian cancer patients (39). This suggests that the omentum is not only a bystander in peritoneal metastases but that it actively participates in ECM dysregulation.

Pancreatic cancer also has the tendency to metastasize to the peritoneum (39) or omentum (208), and Laminin gamma 1 (LAMC1) on pancreatic cancer cells promotes their homing to the peritoneum (30). To gain mechanistic insight into pancreatic cancer propensity to metastasize to the omentum, Feygenzon et al. analyzed the secretome and mRNA expression of tissue explants from human omental fat. These studies revealed higher levels of cancer-related proteins involved in ECM remodeling and ECM-mediated interactions (e.g., TIMP-1, MMP-8, and TSP-1) compared to subcutaneous fat, suggesting that omental fat could reprogram pancreatic cancer cells toward a more aggressive and invasive phenotype, marked by increased levels of ECM proteins and adhesion molecules (208).

LAMC1 is also secreted by gastric cancers, and it accumulated in the pre-metastatic peritoneal microenvironment, where it induced preadipocyte maturation (i.e., differentiation) and altered their metabolism and function, resulting in the release of free fatty acids. Of these fatty acids, palmitic acid regulated LAMC1 in a posttranslational manner and promoted PMN formation and gastric cancer cell colonization of the peritoneum (30).

Finally, the roles of EVs in peritoneal metastasis are only beginning to be explored. Small EVs from malignant ascites of patients with advanced ovarian cancer undergoing adjuvant chemotherapy displayed preferential homing to the peritoneum and were enriched in ECM components and regulatory proteins. These EVs promoted an increase in transforming growth factor-β-I and FNs, which are functionally linked proteins, plasminogen activator inhibitor 1, and FNs, suggesting a role of these EVs in reprogramming peritoneal ECMs toward a pro-metastatic function (209).

## Clinical relevance of PMN ECM remodeling

### Biomarkers of ECM changes

The remote control of PMN formation by the primary tumor secretome presents the opportunity for circulating PMN-inducing biomarker detection and monitoring (MMPs, TIMPs, LOX, and EVs) in liquid biopsies from patients. Indeed, several clinical studies correlated the levels of these circulating biomarkers with clinical outcomes and metastasis, which could be further exploited for the development of therapeutic strategies to impair ECM remodeling that supports PMN formation.

For example, the levels of plasma TIMP-1 can be used as a biomarker, as this soluble tumor-derived secreted factor appears to be the main player in determining the propensity for liver PMN formation in various cancers. Indeed, high levels of plasma TIMP-1 are a poor prognostic indicator for liver metastases (210) and for the progression of gastric (211), colorectal (165), and pancreatic (212) cancers. A recent study identified extravesicular TIMP as a critical mediator of liver PMN reprogramming and ECM remodeling, as well as an independent prognostic factor, associated with poor progression-free survival in patients with colorectal liver metastases (44).

In patients with osteosarcoma, the levels of ANGPTL2, which promotes lung PMN formation, were systemically elevated relative to healthy subjects, thus offering the possibility for intervention to hinder ANGPTL2-mediated signaling and subsequent pro-metastatic effects (213). Importantly, high levels of serum MMPs are also reliable biomarkers indicative of metastatic progression and are associated with poor survival in gastric (MMP-14) (214) and breast (MMP-2 and MMP-9) (215) cancer patients.

The potential use of EVs as biomarkers associated with PMN formation was recently highlighted by Hoshino et al. (216), where unbiased proteomic profiling of EVs from human cancer samples (tumor and adjacent tissue explants, plasma, and other biological fluids) allowed the discovery of pan-cancer EVP markers, with high diagnostic and therapeutic potential (216). Therefore, VCAN, TN-C, and thrombospondin are pan-cancer EVP markers and also ECM components that can discriminate tumors from adjacent tissues with high sensitivity and specificity. Moreover, specific EVP adhesion proteins (e.g., CD36, TN-C, THBS2, and VCAN) may also be pan-cancer markers. Additionally, pancreatic cancer-derived EVs were mainly enriched in ECM-associated proteins, thereby reinforcing the possibility to employ EVs for monitoring the stiffness and changes in ECM deposition in early-stage primary tumors, which may allow early PMN detection (216).

### ECM-targeting therapies

The ECM is a key player in therapeutic resistance (217–219). Structural and physical ECM changes negatively modulate responses to therapies. A dense and rigid ECM protects tumor cells from therapeutic agents, regulating their permeability throughout the microenvironment. Likewise, functional modifications of the ECM can actively interfere with cell cycle arrest, apoptosis, and anti-tumor immunity by sustaining a favorable environment for tumor cells.

ECM remodeling mediated by primary tumors represents one of the first, albeit critical, steps in PMN formation, which offers the advantage to target soluble factors secreted by tumors in systemic circulation to hinder PMN formation or to target PMN-resident or PMN-infiltrating immune and stromal cells that are pivotal for altering ECM homeostasis in distant tissues. However, a word of caution is necessary, as sustained inhibition of ECM may adversely affect normal programs of wound healing, and the timing and duration of ECM-directed treatments in cancer are key (220).

Based on the potential of anti-angiogenic therapies to hinder metastasis and interfere with PMN formation, the use of recombinant circulating anti-angiogenic factors should be further evaluated to determine the therapeutic outcome and PMN formation. A recent meta-analysis showed that recombinant endostatin, Endostar (rh‐endostatin), approved for clinical use in China for almost 20 years, improved the prognosis of non‐small cell lung cancer patients with bone metastases when used in combination with other chemotherapeutic agents (221–223).

For example, pharmaceutical inhibition of ANGPTL2 signaling using a non-RGD-based integrin-binding peptide (ATN-161) could be a potential strategy for interfering with lung PMN formation in osteosarcoma, as suggested by preclinical studies (213). Other preclinical data also show that suppressing the interaction of TIMP-1 with the CD63 receptor on hepatic stellate cells effectively prevented liver PMN formation in pancreatic cancer models (212). Moreover, drugs targeting MMPs (MMP-2 and MMP-9) to interfere with ECM degradation are also promising options for preventing ECM disruption in the PMN (reviewed in 224), especially as there are ongoing clinical trials that target MMPs systemically.

The relevance of targeting LOX proteins systemically or at the PMN is based on their pivotal contribution in regulating ECM remodeling, BMDC docking in the distant organ, and the encouraging preclinical data showing that LOX inhibition reduced hypoxia-induced metastasis (140). Previous therapeutic strategies employing LOX inhibitors that underwent clinical trials, such as LOXL2-blocking monoclonal antibody simtuzumab and small molecule LOXL2 inhibitors (e.g., PAT1251 and PXS-2SA), did not appear to be very effective. Currently, tetrathiomolybdate and pemetrexed AB0024 (anti-LOXL2 monoclonal antibody) are being clinically tested for moderate-to-advanced breast cancer and metastatic non-small cell carcinoma (224). However, these trials are tailored for cancers with established metastases and are not designed to evaluate effects on the PMN. Interestingly, an EGFR inhibitor, WZ4002, in combination with silibinin, was effective in decreasing metastasis in preclinical models by blocking EGFR/LOX pathway and may represent a promising alternative strategy to interfere with LOX-mediated PMN formation (225).

Given the critical role of integrins in mediating organotropic metastases and their central role in ECM-based interactions in the PMN, targeting specific heterodimeric partners either systemically or locally is both a challenging and a promising opportunity. For example, Cilengitide, an RGD pentapeptide targeting both αvβ3 and αvβ5 integrins, has been tested in clinical trials on metastatic and non-metastatic tumors (226). Similar to integrin-targeting strategies, disintegrins, such as vicrostatin, have been tailored to target multiple integrins (i.e., αvβ3, αvβ5, and α5β1), and studies in preclinical cancer models highlighted their translational potential (227). Lastly, small molecules interfering with β1-integrin signaling (e.g., ATN-161, and JSM6427) also showed promise in targeting dormant cancer cells and preventing metastatic recurrence in preclinical models (228).

EVs play a key role in PMN formation, and various therapeutic approaches targeting EVs are currently in development (229). One potential therapeutic strategy would be to interfere with EV production. In preclinical studies, reserpine, an anti-hypertensive drug, could suppress tumor-derived EV uptake and disrupt the EV-induced PMN formation and melanoma lung metastasis, suggesting a potential benefit of reserpine in the adjuvant setting for melanoma treatment (230). Ideally, blocking the delivery of specific EV cargo crucial for ECM remodeling at distant sites could improve patient outcomes. Recently, an interesting therapeutic strategy revealed that the inhibition of HSP90AA on human serum-derived EVs packaging TIMP-1 (which binds HSP90AA) attenuates the ECM remodeling effects of these EVs (44), and this intervention could have a high clinical impact in preventing liver PMN formation. Alternatively, blocking TIMP-1 with monoclonal antibodies should allow the specific systemic targeting of this molecule.

As a large number of studies demonstrated that specific EV integrins selectively adhere to ECM components and ECM-enriched areas in distant pre-metastatic organs, a potential therapeutic strategy would involve active targeting or sequestration of specific circulating EVs with the homing capacity to various PMNs using monoclonal antibodies tailored against integrins (α6β4 and αvβ5) or other proteins (S100A4, VCAN, TNC, and TSP), thereby preventing their intrinsic PMN-promoting roles (35, 216).

In addition to interfering with specific components of the ECM matrisome that promote PMN formation, other potential strategies could aim to target or re-educate stromal or immune cells that are critical for ECM remodeling in specific organs (e.g., CAFs, Kupffer cells, and BMDCs). Thus, recent innovative nano-therapies show translational potential in preclinical models. For example, an anti-thrombotic agent based on calcium phosphate nanoparticles coated with heparin strongly reduced ICAM-1, VCAM-1, and MMP-9 in pre-metastatic lungs of murine breast cancers (231). Drug-free peptide nano-blankets assembled *in situ* interfered with fibroblast activation and subsequent LOX, FN, and periostin accumulation in the early PMN (232).

## Conclusions and perspectives

As opposed to the attention paid to cancer cells, the “soma”, or non-cellular material, has been generally overlooked in the past by oncology researchers. Despite the recent progress in our understanding of the composition and function of the PMN ECM, many outstanding questions remain. For instance, are ECM changes in the PMN reversible, or should therapeutic targeting focus on blocking the already existent PMN-associated ECM alterations? Given the crucial roles of ECM in homeostasis and wound healing, are there cancer-specific/PMN-specific ECM characteristics that can be specifically targeted with minimum side effects, such as particular ECM protein isoforms or post-translational modifications? Since ECM alterations provide a platform for the recruitment of immunosuppressive myeloid subsets, thereby precluding T-cell recruitment or function in the PMN, could disrupting this axis be a promising therapy that could be combined with checkpoint blockade in cancers otherwise refractory to immunotherapy?

To unveil the complete potential of the early detection of ECM pro-metastatic changes (e.g., in patients without metastatic disease) and to identify novel or critical therapeutic targets that are highly specific for PMN formation, a better understanding of ECM molecular and cellular changes in the PMN in cancer patients is needed. Precedent exists in preclinical models, illustrated, for example, by the work of Hebert et al., who conducted a comprehensive, unbiased mass spectrometry analysis of the ECM in metastatic niches such as the brain, lungs, liver, and bone marrow for MDA-MB-231 TNBC (61). This study highlighted the fact that tumor cells and stroma cooperate during metastatic niche formation, with stromal cells building the core, structural matrisome, and tumor cells producing ECM-associated proteins, secreted factors, and ECM regulators. A similar analysis dissecting the PMN matrisome in conjunction with the tumor secretome in patients may unveil novel biomarkers, leading to the discovery of therapies designed to “demolish the nest”, hindering ECM remodeling and preventing PMN formation in patients with early-stage primary tumors. Moreover, using novel innovative techniques such as MicroMap in PMN preclinical models and patient tissue samples may enable the discovery of novel, PMN-specific ECM–receptor interactions that could be targeted in the clinic (233).

From a therapeutic perspective, preclinical studies often highlight the possibility of using both novel therapies currently under clinical investigation as well as repurposing already Food and Drug Administration (FDA)-approved therapies that could interfere with all aspects of ECM-associated changes in distant organs. However, clinical trials that include investigation of PMN or ECM-associated changes at future metastatic sites are relatively rare, despite the availability of a number of non-invasive clinical tools that could reveal PMN formation. This impedes the identification and clinical validation of PMN/ECM-associated markers that could be detected early on during the cancer progression timeline, before metastatic onset. As an example, the use of new technologies or existing imaging techniques, such as the ultrasound-based FibroScan or artificial intelligence-assisted computer tomography, may help detect structural or quantitative ECM changes such as fibronectin deposition, tissue stiffness, and fibrotic environments, which occur in the lung, liver, and LNs, and therefore guide therapeutic decisions. Our overview of the current knowledge of ECM biology, often taken for granted or dismissed as peripheral, highlights its pleiotropic roles at each step of the metastatic cascade and its theranostic as well as therapeutic potential.

## Author contributions

All authors listed have made a substantial, direct, and intellectual contribution to the work and approved it for publication.
